# Gamma knife radiosurgery for recurrent gliomas

**DOI:** 10.1007/s11060-018-2988-0

**Published:** 2018-09-06

**Authors:** Zjiwar H. A. Sadik, Patrick E. J. Hanssens, Jeroen B. Verheul, Guus N. Beute, Suan Te Lie, Sieger Leenstra, Hilko Ardon

**Affiliations:** 1 Gamma Knife Center, Elisabeth-Tweesteden Hospital, Tilburg, The Netherlands; 2 Department of Neurosurgery, Elisabeth-Tweesteden Hospital, Tilburg, The Netherlands; 3 Department of Neurosurgery, Amsterdam Medical Center, Amsterdam, The Netherlands; 4 Department of Neurosurgery, Erasmus Medical Center, Rotterdam, The Netherlands

**Keywords:** Gamma knife radiosurgery, Recurrent, Gliomas

## Abstract

**Objective:**

In recent years, gamma knife radiosurgery (GKRS) has become increasingly more popular as a salvage treatment modality for patients diagnosed with recurrent gliomas. The goal of GKRS for recurrent glioma patients is to improve survival rates with minimal burden for these patients. The emphasis of this report is on local tumor control (TC), clinical outcome and survival analysis.

**Methods:**

We performed a retrospective analysis of prospectively collected data of all patients who underwent GKRS for gliomas at the Gamma Knife Center Tilburg between 23-09-2002 and 21-05-2015. In total, 94 patients with glioma were treated with GKRS. Two patients were excluded because GKRS was used as a first stage treatment. The other 92 patients were included for analysis.

**Results:**

TC was 37% for all tumors (TC was 50% in LGGs and 27% in HGGs). Local progression (LP) was 46% for all tumors (LP was 31% in LGGs and 58% in HGGs). New distant lesions were seen in 18% of all patients (in 5% of LGG patients and 31% of HGG patients). Median progression-free and overall survival (PFS and OS) for all patients were 10.5 and 34.4 months, respectively. Median PFS was 50.1 and 5.7 months for low and high grade tumors, respectively. Median OS was 86.6 and 12.8 months for low and high grade tumors, respectively. No serious adverse events were noted post-GKRS.

**Conclusion:**

GKRS can safely be used as salvage treatment for recurrent glioma and seems to improve survival rates in (high grade) glioma patients with minimal burden.

## Introduction

Gliomas are the most common primary malignant brain tumors. The overall age-adjusted incidence rates for all gliomas range between 4.67–5.73/100,000 persons/year [1, 2]. Gliomas have been classified into four grades of ascending malignancy by the World Health Organization (WHO). Essentially, they can be divided into two major groups: low grade gliomas (LGG; WHO 1–2) and high grade gliomas (HGG; WHO 3–4). GBM (WHO grade 4) is the most deadly glioma in adults with an overall 5 year survival of 0.05–4.7% [3–7]. Generally, gliomas are more common in men than in women [3, 8–10]. Currently, the treatment of gliomas consists of maximal safe surgical resection, external beam radiation therapy (EBRT), and chemotherapy. However, these tumors do often reoccur. Options for salvage treatment are repeated surgery, re-irradiation with EBRT, chemotherapy, novel therapies, or a combination of these treatments. Repeated surgery can be a good option as a salvage treatment, but might be associated with postoperative complications. Treatment with EBRT for a second time can be accompanied by high risk of radiation-related toxicity and necrosis. In recent years, gamma knife radiosurgery (GKRS) has become increasingly more popular as a salvage treatment modality for patients diagnosed with recurrent gliomas. The goal of GKRS for recurrent glioma patients is to improve survival rates with minimal burden for these patients. We summarize our experience in a group of 92 patients with recurrent glioma, treated with GKRS. The emphasis of this report is on local tumor control (TC), clinical outcome and survival analysis.

## Methods

### Case selection

We performed a retrospective analysis of prospectively collected data of all patients who underwent GKRS for gliomas at the Gamma Knife Center Tilburg between 23-09-2002 and 21-05-2015. The study was approved by the medical ethical committee. Hospital records, including clinical notes, doctors’ letters, radiology reports and demographic data, were reviewed and relevant information was extracted for analysis. Pre- and post-GKRS clinical characteristics were reviewed. We included all patients with histologically confirmed gliomas. In total, 94 patients with glioma were treated with GKRS. Two patients were excluded because GKRS was used as a first stage treatment. The other 92 patients were included for analysis.

### Baseline characteristics

As stated, 92 patients (52 males and 40 females) underwent GKRS for recurrent glioma. The median age at time of GKRS was 50 years (range 7–76) **(**Table 1**)**. The histopathology of the tumors is summarized in Table 2. Eighty-five patients had undergone at least one operation before GKRS. Seven patients received biopsy followed by adjuvant treatment in the form of chemotherapy, EBRT or a combination of both, before GKRS. Pre-GKRS treatment features are summarized in Table 1.

**Table 1:** Pre-GKRS patient characteristics and radiosurgical features

Patient characteristics	No. of patients (%)
Total patients included	92
Male: female ratio	52:40
Median age (range)	50 (7–76)
Median Karnofski index (range)	90 (50–100)
≥ 70	80
< 70	12
Histopathology	
WHO I glioma	17 (19%)
WHO II glioma	26 (28%)
WHO III glioma	24 (26%)
WHO IV glioma	25 (27%)
Primary surgery	
Complete resection	78 (84%)
Subtotal resection	7 (8%)
Biopsy	7 (8%)
Adjuvant therapy	
Repeated surgery	1 (1%)
PVC + EBRT + adjuvant TMZ	26 (28%)
EBRT + TMZ	2 (2%)
EBRT only	40 (43%)
Target location	
Infratentorial	19 (18%)
Supratentorial	88 (82%)
Unifocal	79 (86%)
Multifocal	13 (14%)
Median tumor volume in cm^3^ (range)	2.22 (0.01–9.95)
Radiosurgical features	
Median PD in Gy (range)	18 (12–25)
Median minimal dose in Gy (range)	17.7 (9.4–26.1)
Median maximal dose in Gy (range)	34.6 (19.4–51.2)
Median isodose in % (range)	52 (39–93)
Median coverage in % (range)	99 (89–100)
Median no. of lesions treated (range)	1 (1–4)

*EBRT* External beam radiotherapy, *PD* Prescribed dose, *PVC* Procarbazine, Lomustine and vincristine, *TMZ* Temozolomide

**Table 2:** Histopathology and radiosurgical response according to the Tilburg radiological classification

Histopathology	No. of patients	No. of tumors	TC	LP	MP	NDL
WHO I						
Pilocytic astrocytoma	10	10	7	3		
Subependymoma	4	4	2	1	1	
Myxopapillary ependymoma	1	2	2			
Ganglioglioma	1	1	1			
Subependymal giant cell astrocytoma	1	1	1			
WHO II						
Oligodendroglioma	11	12	3	9		5
Ependymoma	10	13	11		1	7 + 1 Lepto
Astrocytoma	2	2	1	1		
Oligoastrocytoma	2	2		1	1	1
Central neurocytoma	1	1	1			1
WHO III						
Anaplastic oligodendroglioma	11	15	4	6	3	5
Anaplastic astrocytoma	7	10	4	3	2	4
Anaplastic ependymoma	3	3	1	2		
Anaplastic oligoastrocytoma	3	3		2	1	1
WHO IV						
Glioblastoma multiforme	25	28	7	21	1	5
Total	92	107				

*Lepto* Leptomeningeal, *LP* Local progression, *MP* Marginal progression, *NDL* New distant lesion, *TC* Tumor control

### Gamma knife radiosurgical procedure

The GKRS procedure was performed using the Leksell Gamma Knife 4C, before 2009, and Leksell Gamma Knife Perfexion thereafter. GammaPlan Software (Elektra) was used for treatment planning. The application of the Leksell G-Frame with fixation posts and screws at four points was performed at the patients room using a local anaesthetic solution (9 ml of lidocaine 2% + epinephrine 0.125% combined with 1 ml of NaHCO_3_ 84 g/1000 ml). Following frame placement, high resolution stereotactic MRI was performed for treatment planning. Pre- and post-contrast (Triple dose Gadolinium) T1 weighted axial images were obtained with a slice thickness of 1.5 mm. Stereotactic radiosurgery and dose planning were then performed in consultation with a neurosurgeon, radiation-oncologist, and medical physicist. The target was defined as the contrast enhancing lesion on the planning MRI-scan. Target delineation was limited to the target enhancing lesion only for progressive LGG (WHO I and II) and HGG (WHO III and WHO IV) as confirmed by the ASTRO guidelines [11]. The median number of lesions treated with GKRS was 1 (range: 1–4) with a median tumor volume of 2.22 cm^3^ (range: 0.01–9.95). Eventually, a total of 107 (48 LGG and 59 HGG) tumors were treated with GKRS. The prescribed dose (PD) ranged between 12 and 25 Gy (median 18 Gy) to that isodose covering 89–100% of the target **(**Table 1**)**.

The median PD for LGG and HGG was 18 Gy ranging between 12 and 25 Gy and 14–25 Gy, respectively. After GKRS, patients received 8 mg dexamethasone for 5 days and thereafter the dose was decreased to 0 mg within 1 week.

### Radiological classification for progression

**Fig. 1:**
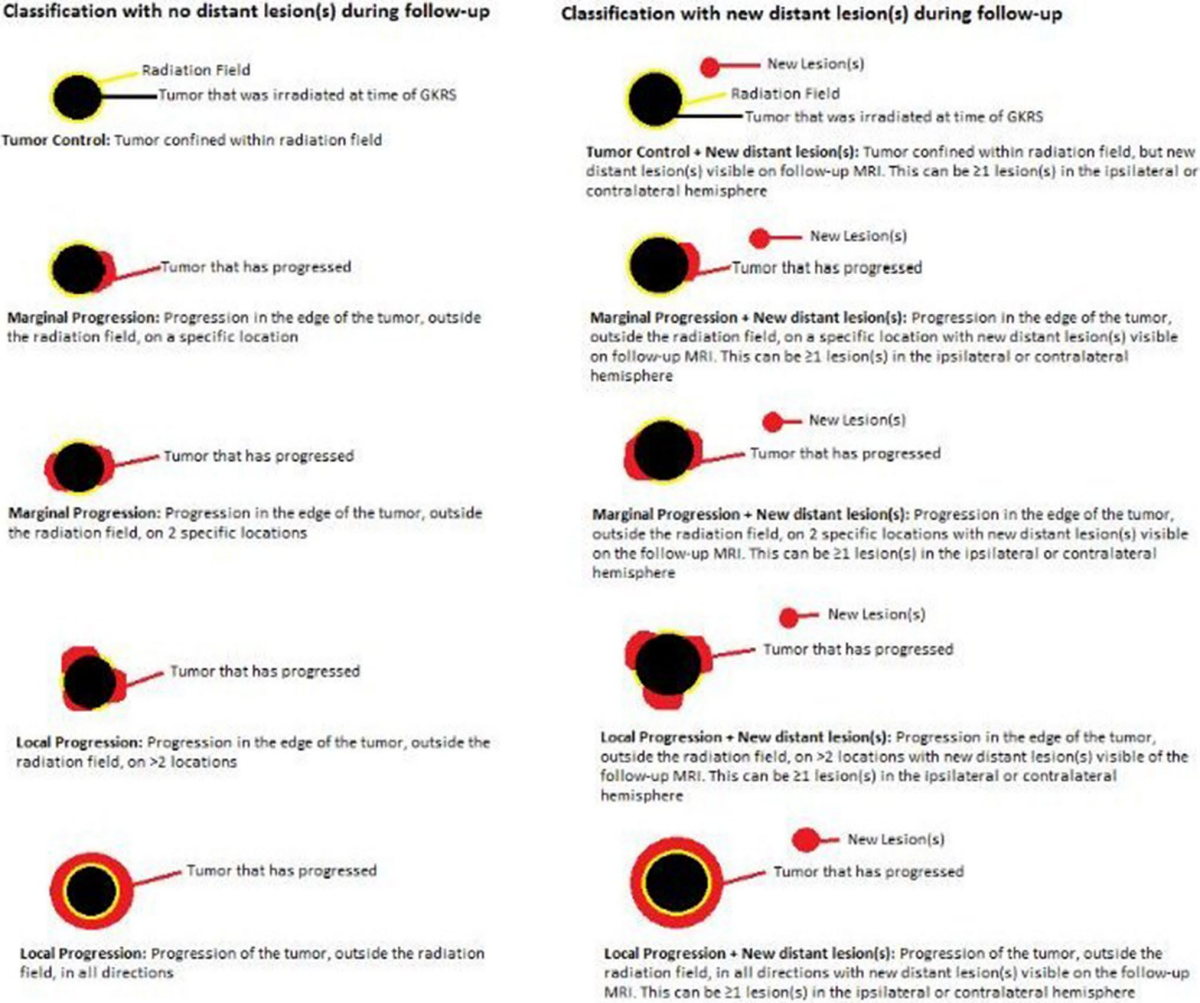
Tilburg radiological classification

Determining radiological progression (after treatment) is a difficult task for gliomas, especially differentiating between progression/recurrence and treatment induced radiological changes. Therefore, we have introduced the Tilburg radiological classification for scoring TC after GKRS. Figure 1 shows the Tilburg classification on which we based our definitions of tumor progression. A subdivision was made between local TC with or without (a) new distant lesion(s).

### Statistical analysis

Data are represented as median with the range. The Pearson correlation coefficient was obtained through linear regression analysis. Survival analysis was done by log rank test on Kaplan Meier survival estimates. Survival was defined as time from GKRS till death or last FU. Statistics were calculated with Prism software 7 (GraphPad Software Inc., San Diego, CA, USA).

## Results

### Radiosurgical response/radiological tumor control

TC was achieved in 37% of all tumors treated. According to tumor grade, TC was obtained in 50% of LGGs (24 out of 48 tumors) and in 27% of HGGs (16 out of 59 tumors). Local progression (LP) occurred in 46% of all tumors, in 31% of LGGs (15 out of 48 tumors) and in 58% of HGGs (34 out of 59 tumors). Marginal progression occurred in 9% of all tumors, in 6% of LGGs (3 out of 48 tumors) and in 12% of HGGs (7 out of 59 tumors). New distant lesions (NDL) were seen in 18% of all patients; in 5% of LGG patients (2 out of 43 patients) and 31% of HGG patients (15 out of 49 patients) **(**Table 2**)**. One (LGG) patient had leptomeningeal disease **(**Table 2**)**.

### Survival and prognostic factors/clinical outcome

**Fig. 2:**
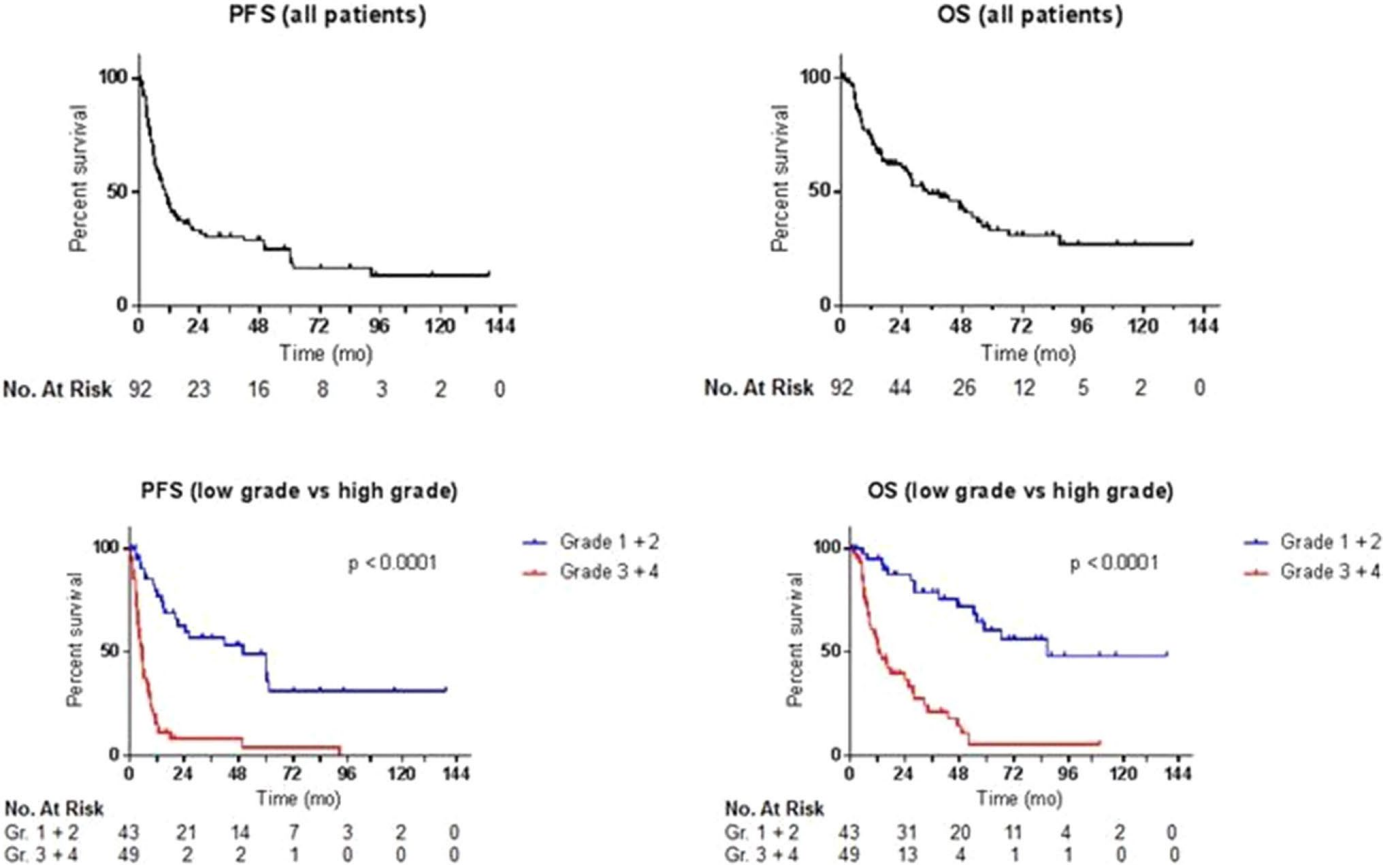
Progression-free and overall survival (PFS and OS) for all patients and according to tumor grade (LGG vs. HGG). Median PFS was 10.5 months (range 0.4–139.4 months) and median OS was 34.4 months (range 0.5–139.4 months). (*mo* months). Low grade tumors were defined as grade 1 and 2 tumors, high grade tumors as grade 3 and 4 tumors. Median PFS was 50.1 months (range 1.3–139.4 months) and 5.7 months (range 0.4–92.5 months) for low and high grade tumors respectively. Median OS was 86.6 months (range 1.3–139.4 months) and 12.8 months (range 0.5–109.5 months) for low and high grade tumors respectively. (*mo* months, *vs* versus)

Kaplan Meier survival curves are given in Fig. 2. Median progression-free and overall survival (PFS and OS) for all patients were 10.5 and 34.4 months, respectively **(**Fig. 2**)**. Median PFS was not yet reached for grade 1 (range 7.7–139.4 months), 14.3 months for grade 2 (range 2.8–139.1 months), 8.8 months for grade 3 (range 1.5–49.6 months) and 4.4 months for grade 4 glioma (range 0.4–18.1 months). Median OS for the aforementioned tumor grades was not yet reached (range 7.7–139.4 months), 47.2 months (range 2.8–139.1 months), 18.1 months (range 3.3–52.0 months) and 10.4 months (range 1.4–32.7 months), respectively. There was a highly significant increase in both PFS and OS according to tumor grade; i.e. the lower the tumor grade, the better the outcome **(**Fig. 2**)**. Low grade tumors were defined as grade 1 and 2 tumors, high grade tumors as grade 3 and 4 tumors. Median PFS was 50.1 months (range 1.3–139.4 months) and 5.7 months (range 0.4–92.5 months) for low and high grade tumors, respectively. Median OS was 86.6 months (range 1.3–139.4 months) and 12.8 months (range 0.5–109.5 months) for low and high grade tumors, respectively **(**Fig. 2**)**. There was no correlation between time from first diagnosis to GKRS and PFS. The median follow-up (FU) was 34 months from the date of GKRS treatment. We lost 56 patients during FU. All of these patients died due to intracranial progressive disease.

### Adverse events

Seventy-nine percent (36 out of 48) of the LGG patients had no adverse events. Frame related swelling occurred in 10% (5 out of 48) of the patients within a median time of 14 days **(**Table 3**)**.

**Table 3:** Adverse events after radiosurgery

Adverse events^a^	LGG	Median time to occur	HGG	Median time to occur
None	36 (79%)		35 (59%)	
Frame related swelling/pain	5 (10%)	14 days	5 (9%)	28 days
Headache (grade 1)	0		3 (5%)	28 days
Headache (grade 2)	2 (4%)	84 days	1 (2%)	28 days
Sensitive headskin (grade 1)	1 (2%)	14 days	1 (2%)	56 days
Focal alopecia (grade 1)	1 (2%)	21 days	0	
Concentration loss (grade 1)	1 (2%)	56 days	0	
Fatigue (grade 1)	1 (2%)	9 days	3 (5%)	28 days
Dizziness (grade 1)	1 (2%)	56 days	0	
Vertical diplopia (grade 1)	1 (2%)	6 months	0	
Somnolence (grade 1)	1 (2%)	18 days	0	
Focal epilepsy (grade 1)	1 (2%)	20 days	3 (5%)	1 day

^a^CTCAE (CTC-M) version 4.0 (common terminology criteria for adverse events)

In HGG patients, 59% had no adverse events. In this group the incidence of frame related swelling was comparable to that of LGG patients. Other adverse events for both LGG and HGG patients are summarized in Table 3. No serious adverse events were noted after GKRS **(**Table 3**)**.

## Discussion

In recent years, GKRS has become increasingly more popular as a salvage treatment modality for patients diagnosed with recurrent gliomas. The goal of GKRS for recurrent glioma patients is to improve survival rates with minimal burden for these patients. One can argue about what type of treatment is best for patients with recurrent glioma. This is dependent on several factors, such as patient’s age, comorbidity, Karnofski-Index, histopathological diagnosis and available treatment modalities. In our center, a difference is made between low grade and high grade tumors. Patients with recurrent low grade tumors are more suitable to undergo reoperation because these tumors are less responsive to radiosurgery. However, this is dependent on location and tumor volume. Patients with recurrence in eloquent locations and/or with small tumor volume are less suitable to undergo reoperation and are more suitable candidates to undergo minimal invasive treatment such as GKRS. In case of high grade tumors, patients with recurrence have limited survival [12–14] and are more eligible for GKRS. Moreover, high grade tumors have a better radio-surgical response in comparison to low grade tumors. Finally, for patients with glioma the quality of life (QoL) is an increasingly important recognized factor. Not only do these patients suffer from general symptoms associated with cancer, such as fatigue, anxiety, and depression, but also from seizures, cognitive deficits, and focal neurological deficits. Therefore, treatments leading to deterioration of QoL are less favorable for these patients, giving their short lifespan, making GKRS an excellent treatment modality for maintaining QoL and extending survival time.

Determining radiological progression is known to be difficult for gliomas, especially after different treatment modalities have been applied. Until recently, clear definitions of progression and pseudo-progression have been lacking, which makes comparison between different studies on treating recurrent glioma bothersome. For this, the RANO (Response Assessment in Neuro-Oncology) criteria have been proposed to score radiological (pseudo-) progression more uniformally. However, these RANO criteria have not specifically been designed for patients treated with GKRS. Therefore, we have introduced the Tilburg radiological classification for scoring TC after GKRS. The primary goal of this classification is to allow us to compare our own treatment results more objectively. Furthermore, this classification could form the basis for uniform radiological criteria scoring radio-surgical response.

In our study, TC was achieved in 37% of all tumors; 50% in LGGs and 27% in HGGs. A recent study showed a 53.8% TC for LGGs after GKRS [15], which is comparable to our study. For HGGs, the TC is within range of other studies which reported TC of 18.8%–75.6% [16–19]. LP occurred in 46% of all tumors treated with GKRS. This might indicate a dose problem, meaning that a higher dose is needed to obtain a higher local TC. Another important subject is target definition which led to marginal progression in 9% of all tumors in our study. Koga et al. were the first to address these two problems [19]. They compared two groups, conventional SRS (20 Gy applied to the margin of each gadolinium-enhanced lesion) versus extended field SRS (margin was extended up to 0.5–1 cm). TC in the conventional group was 47%. By extending the margin of the clinical target volume the TC increased to 93% (P = 0.0035). However, there was no significant difference (P = 0.83) in survival time between the two groups. A higher incidence of radiation necrosis was seen in the extended SRS group (29% vs. 6.5%). The goal of extending the irradiation field is to include as many tumor cells invading the surrounding tissue as possible. A limitation of this method is that it is not applicable to large lesions. Extending the margin in a large target results in a large prescribed isodose volume and might cause uncontrollable radiation induced adverse events. In case of GKRS as salvage treatment, the goal is prolonged survival with minimal burden. Therefore we do not find it useful to irradiate more than the enhanced lesion because of the higher incidence of radiation necrosis and uncontrolled radiation induced adverse events. Gliomas are highly vascular tumors and have a tendency to infiltrate extensive areas of the brain. The real tumor may therefore be much larger than what is seen on MRI, making a 0.5–1 cm margin still insufficient. This is another reason to only irradiate the enhanced lesion for both high and low grade gliomas.

The median PFS and OS for all patients were 10.5 and 34.4 months, respectively. Median PFS was 50.1 months and 5.7 months for low and high grade tumors respectively. Median OS was 86.6 months and 12.8 months for low and high grade tumors respectively. This is comparable to other studies about GKRS for recurrent HGG who showed a median OS ranging from 13 to 14 months [18, 20–22].

Other studies in which radiosurgery was performed using a linear accelerator (LINAC) for the treatment of recurrent HGG showed comparable median OS ranging from 8.5 to 11 months [23–25]. As could be expected, there was a highly significant increase in both PFS and OS according to tumor grade (i.e. the lower the tumor grade, the better the outcome), reflecting the natural behaviour of these tumors and sensitivity to treatment. Of clinical interest, in recurrent GBM patients median OS was 10.4 months. This shows that GKRS can meaningfully lengthen survival in these patients with a 1 day hospitalization, maintenance of QoL and minimal burden. For patients with a limited life span, these advantages should make one consider GKRS as a treatment option in recurrent glioma patients, as opposed to systemic treatment modalities that might hamper QoL (and might increase the number of hospital visits needed for therapy).

It is important to note that there were no serious adverse events and that the therapy was well tolerated. In general, only mild adverse events were noticed and GKRS was well tolerated.

In the Netherlands, the first line of therapy for HGG is surgical resection followed by chemo-radiation [26]. In case of recurrence, several options do exist: repeat surgery, RT, chemotherapy, experimental therapy or a combination of these treatment modalities, all depending on the clinical state of the patient. We are one of only two centers in the Netherlands who have access to GKRS. In case of recurrent glioma we can use high single dose radiation for small (multiple) lesions without causing damage to surrounding brain tissue, which already has been exposed to radiation. This is a clear advantage over conventional RT. Therefore, our recommendation is to use GKRS if possible in small recurrent glioma since one can give a higher dose radiation in an already irradiated brain compared to conventional RT, minimizing the possible risk of side-effects.

We use triple dose gadolinium for the planning MRI-scan in combination with a small slice thickness of 1.5 mm. This allows for detection of very small recurrence lesions. This is another advantage of using GKRS, because we can treat lesions in a very early stage of recurrence.

## Limitations of the study

This is a single-center retrospective study with a relatively small number of patients and therefore subjected to biases (selection bias and treatment bias) and limitations. The recurrent glioma volume that was treated was rather small, as a result of selection bias. The patients that are eligible for GKRS treatment, are patients with smaller tumor volumes. As a result, the patients that are treated with GKRS probably have a less unfavourable prognosis. All patients were pre-treated before GKRS. Major limitations are non-protocolized treatment regiments. The treatment included multiple surgeries, use of chemotherapeutic agents, different radiotherapy regimens, and heterogeneity in primary brain tumors. Therefore the group we present is very heterogeneous, which makes comparison to historical groups very difficult.

## Conclusion

GKRS can safely be used as salvage treatment for recurrent glioma and seems to improve survival rates in (high grade) glioma patients with minimal burden.
